# Supplementary material for: The causal role of circulating inflammatory markers in osteoporosis: a bidirectional Mendelian randomized study
Source: Front Immunol. 2024 Jul 18;15:1412298. doi: 10.3389/fimmu.2024.1412298 (PMC11291241; doi:10.3389/fimmu.2024.1412298)
Supplement: Supplementary file 1 [file DataSheet_1.zip › supplementary material/CIM-OSTEOPOROSIS /GCST90274832.csv_forest.pdf]

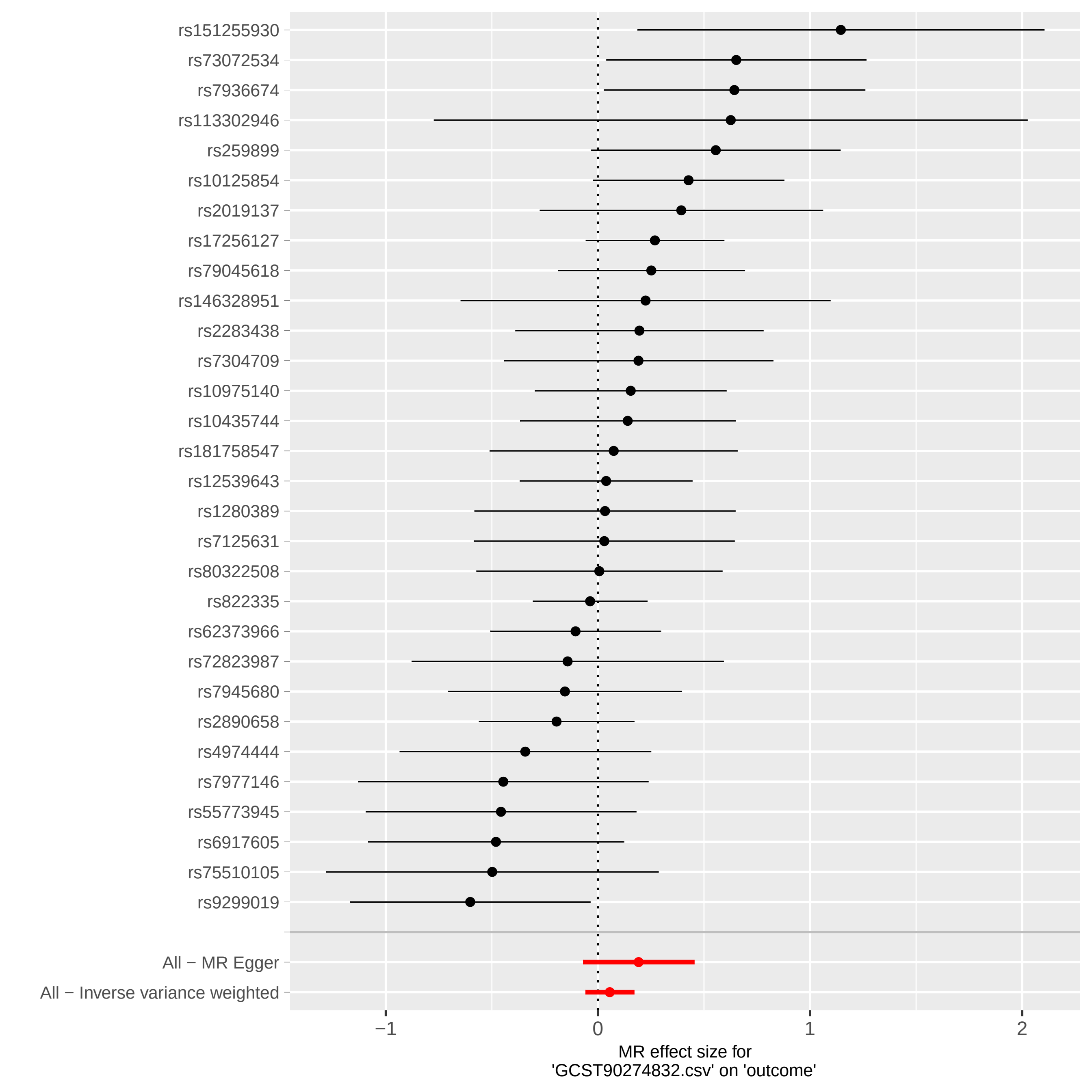
rs151255930
rs73072534
rs7936674
rs113302946
rs259899
rs10125854
rs2019137
rs17256127
rs79045618
rs146328951
rs2283438
rs7304709
rs10975140
rs10435744
rs181758547
rs12539643
rs1280389
rs7125631
rs80322508
rs822335
rs62373966
rs72823987
rs7945680
rs2890658
rs4974444
rs7977146
rs55773945
rs6917605
rs75510105
rs9299019
All − MR Egger
All − Inverse variance weighted
−1
0
1
2
MR effect size for
'GCST90274832.csv' on 'outcome'
